# Osteotome sinus floor elevation without grafting material: Results of a 2-year prospective study

**DOI:** 10.4317/jced.51576

**Published:** 2014-12-01

**Authors:** Aritza Brizuela, Nerea Martín, Felipe J. Fernández-Gonzalez, Carolina Larrazábal, Alberto Anta

**Affiliations:** 1 DDS,PhD,MsC. Professor, Postgraduate Department of Oral Implantology, School of Medicine and Dentistry, University of the Basque Country, Leioa, Spain; 2 DDS. Professor, Department of Integrated Adult Dentistry, Department of Stomatology, School of Medicine and Dentistry, University of the Basque Country, Leioa, Spain; 3 BSD, MSD, Specialist in Orthodontics and Dentofacial Orthopedics at the University of Oviedo. Private practice, León, Spain; 4 DDS. Professor, Postgraduate Department of Oral Surgery. Universidad Católica de Valencia. Valencia, Spain; 5 DDS,PhD,MsC. Professor, Postgraduate Department of Oral Implantology, School of Medicine and Dentistry, University of the Basque Country, Leioa, Spain

## Abstract

Objectives: The aim of this prospective clinical trial was to evaluate the success implant rates during 24 months using OSFE procedure without grafting materials. Study design: 42 adult patients (22 female, 15 male) were selected according to Nedir et al´s inclusion criteria of which 5 patients were excluded, due to periapical pathology in adjacent teeth (n=3) and treatment with bisphosphonates (n=2). 37 patients aged 31-68 years were selected. Smokers were divided in two groups depending on the number of cigarettes consumed per day (a) 0-10, (b) 11-20. One patient was excluded because he was lost to follow-up at 24 months A total of 36 threaded implants were placed, ∅4,1mm Straumann® (Straumann AG, Waldenburg, Switzerland) and ∅3,5mm Klockner® (Klockner Implant System, Barcelona, Spain). The most used implant diameter was 4,1 mm (n=29), followed by 3,5 mm (n=7), and length used was 10 mm (n=32) and 8 mm (n=4). Initial RBH ranged from 4 mm to 9 mm. All statistical data were processed using the program R 3.0.2 for windows. Results: A total of 36 threaded implants were placed. Residual bone height (RBH) at implant placement averaged 7,4 ± 0,4 mm. Mean bone gain was 1,8 ± 0,3 mm. Four implants showed a bone gain exceeding 3 mm. Mean implant protrusion length into the sinus amounted to 2.1 ± 0,3 mm. Regarding the relationship between smoking and periodontal probes, no statistically significant differences were found (P=0,25), neither in relation to the number of threads that the implants showed (P=0,29) or bone gain (P=0,79). After 24 months the implant success rate was 91,6%. Conclusions: Implant rehabilitation of edentulous atrophied posterior maxilla can be safely performed and simplified using the OSFE technique without grafting with reliable long-term results.

** Key words:**Crestal bone loss, dental implants, internal sinus lift, no grafting, osteotome sinus elevation, grafting, sinus floor elevation.

## Introduction

The posterior maxilla is often a complicated area for implant placement due to low quality or quantity of bone. To increase the amount of bone and improve the implants fixation, it is usually performed maxillary sinus floor elevation techniques which actually are predictable procedures that allow the creation of new bone improving primary stability and future osseointegration.

The two main ways to access the maxillary sinus cavity in order to elevate Schneiderian membrane are: The lateral approach that it is the most known despite being, invasive, complicated and long-lasting procedure (1,2) and the osteotome sinus floor elevation (OSFE) which was first introduced by Tatum (1986) (3). In its origins it was performed with a special instrument known as a ‘socket former’ which was used to infracture the sinus floor and to move it in a more apical direction. Later, another transalveolar technique, the bone-added osteotome sinus floor elevation, was described by Summers (1994) (4). OSFE is less invasive, traumatic and time consuming than the lateral approach (5). 

The necessity of sinus grafting in the OSFE technique is still in continuing debate, on the one hand according to the original Summers’ publication autogenous, allogenic or xenogenic grafting materials are often filled into the elevated area to maintain the space for new bone formation. Pjetursson *et al.* in their study (6) evaluated the radiographic tissue remodeling of OSFE with or without grafting, and concluded that OSFE should be performed in conjunction with the application of bone or bone substitute grafting material if optimal outcomes were expected. However, the patients were not randomly assigned, and significant inter-group difference in initial bone height was found before surgery so the results of new bone formation couldn´t be compared. On the other hand, there were also found positive results in OSFE procedure without grafting material (7-13).

The objective of this prospective clinical trial was to assess the survival and success implant rates during 24 months using OSFE technique without grafting materials.

## Material and Methods

This study is a prospective clinical trial. The study design and clinical procedures were performed in accordance with Helsinki Declaration revised in 2008. All patients were in good health and were informed about the risks and benefits that the surgery entailed and signed the informed consent form before treatment. The follow-up period was 2 year.

Nedir *et al.* inclusion criteria with small changes were performed to enroll in this study (7,8).

. Patients who need implant treatment in the posterior maxilla.

. The OSFE procedure performed without grafting material.

. 10-mm long implants were planned, and shorter ones (6 and 8 mm) were admitted only in case of membrane perforation.

. Residual bone height (RBH) of ≤9 mm on mesial or distal implant side.

. Bone >1 mm was required on mesial and distal sides to ensure implant stability.

. Implants had to penetrate at least 1mm into the sinus on mesial or distal implant side.

. Implant primary stability had to be achieved.

. Patients agreed not to wear a removable partial denture during the healing period.

All patients who had acute or chronic infectious/inflammatory processes in the maxillary sinus as well as those with periapical disease in adjacent teeth or those in treatment with bisphosphonates were excluded from the study.

According to these criteria 42 adult patients were evaluated, of which 5 patients were excluded, due to periapical pathology in adjacent teeth in 3 cases and treatment with bisphosphonates in the other 2 subjects. 37 patients (22 female, 15 male) aged 31-68 years (mean 56,09 years) were selected. One patient with an implant was excluded because he was lost to follow-up at 24 months. A total of 36 threaded implants were placed 29 | 4,1mm Straumann® (Straumann AG, Waldenburg, Switzerland) and 7 | 3,5mm Klockner® (Klockner Implant System, Barcelona, Spain). Smokers were divided in two groups depending on the number of cigarettes consumed per day, the first group was composed by smokers around 1-10 cigarettes, the second one around 11-20 and those on over 20 cigarettes a day, were excluded from the study. All of them accepted 12-monthly checks and follow-up with X-rays.

Initial RBH ranged from 4 mm to 9 mm. The teeth most replaced were the first upper molars followed by the second premolars. All statistical data were processed using the program R 3.0.2 for windows.

The survival criteria proposed by Buser *et al.* (1997) and Cochran *et al.* (2002) (14,15) were used including: (a) absence of clinically detectable implant mobility, (b) absence of pain or any subjective sensation, (c) absence of recurrent peri- implant infection and (d) absence of continuous radiolucency around the implant.

**Figure 1:**
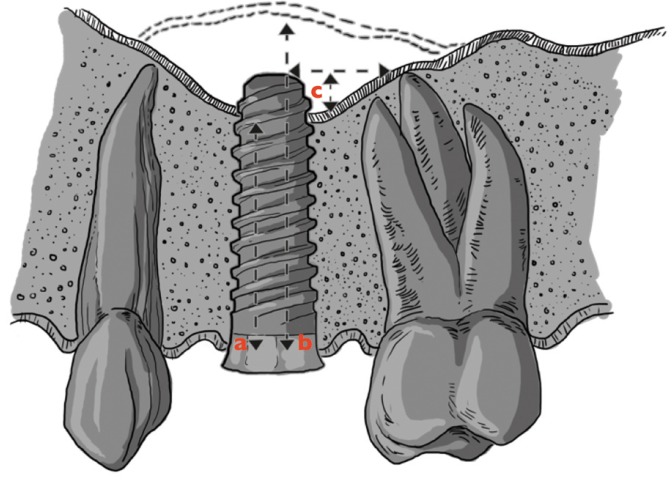
a = RBH (residual bone height). b-a = Bone Obtained. c = protussion length.

For standardization, the same film holder-beam device was employed. The radiographs were taken with the film placed parallel to the implants and the X-ray beam directed perpendicular to the implants. For better reproducibility, the implant suprastructure and the incisal edges of the neighboring teeth were taken with an impression material. It resulted in a device in order to improve reproducible repositioning. Radiographs were taken pre and post-implant placement, every 12 months until 2 years. Implant measure was used as calibration tool (10 and 8 mm). The radiographic analysis was performed by an investigator not involved in the surgical procedure. The following parameters were recorded: a) RBH at implant placement, b) Implant protrusion length, c) Implant crestal bone levels and d) Implant sinus amount of bone (Fig. 1).

## Surgical Technique

The same surgical procedure was used in all subjects, being performed by the same surgeon, auxiliary staff, instruments and equipment. 48 hours before surgery, pattern in antibiotic prophylaxis was introduced by amoxicillin (750mg, tablets) with a regimen of 1 pill every 8 hours. In allergic to penicillin was replaced by Diacetyl-midecamycin (600mg, tablets) 1 pill every 12 hours.

Prior to anesthesia, a mouth rinse with chlorhexidine 0.12% was carried out for one minute. Infiltrative anesthesia was performed using 4% articaine with 1:100.000 epinephrine.

In all cases, a midcrestal incision was performed, separating the keratinized gingiva available and intrasulcular of the adjacent teeth, extra vertical release incisions were not necessary, in order to elevate a full thickness flap.

**Figure 2:**
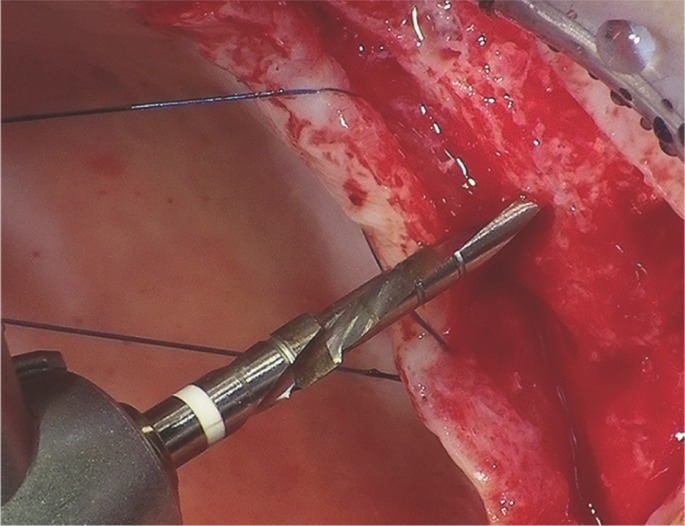
A 1.8mm helicolidal bur.

**Figure 3:**
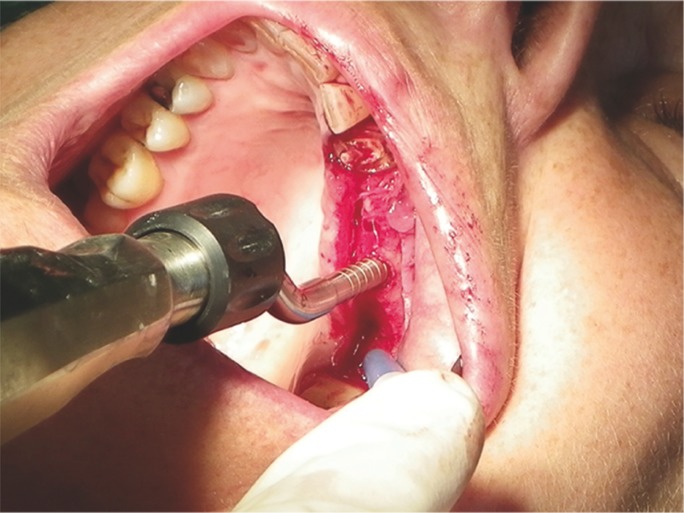
Cortical fracture performed with an osteotome.

Then it was come to the ostectomy with a helicolidal bur of 1.8mm (Fig. 2), up to 1mm of the sinus floor cortical. Thereafter, the fracture of the cortical was performed with prior diameter osteotome of the implant (Fig. 3), in order to finish of forming the bed with an osteotome with the same diameter as implant, which also elevated the membrane to the final working depth (length of the implant), without the placed of any grafting material. The implants were placed through a programmed motor with 35N of maximum torque. A transepithelial screw, 1mm greater than the thickness patient gingival biotype then flap was repositioned and sutured by two single points on each side of the screw by 4 zeros nylon nonabsorbable suture.

**Figure 4:**
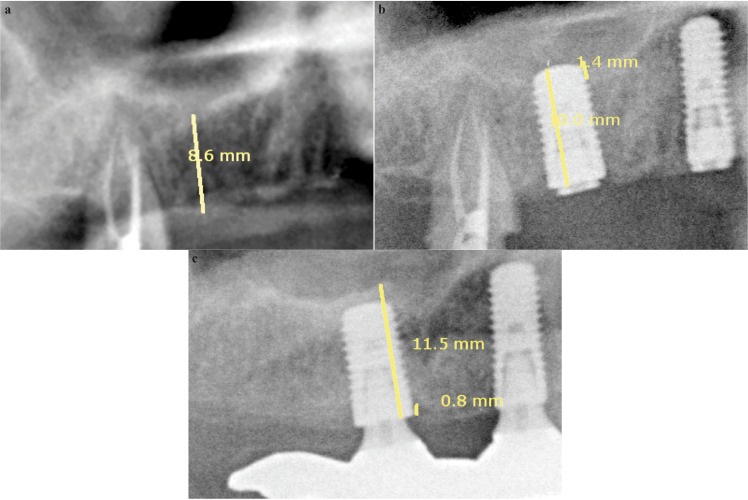
Radiographic evolution of sinus demarcation: a) before surgery, b) Year 1 and c) Year 2.

Antibiotic treatment was established with the pattern described above for 4 days. Ibuprofen (600mg) every 8 hours during 2 days also applications of 0.12% chlorhexidine gel after meals, until suture removal at 10 days. Cicatrization of the bone was controlled along with the taking of the implants by six-monthly until two years x-rays (Fig. 4).

## Results

After clinical and x-ray control of all the patients the results were as follows. 36 threaded implants were placed; 23 were placed in the molar area and 13 in the premolar. Results are shown in  Table 1. Twenty-seven implants were 10 mm long. Membrane perforation led to placement of four 8 mm long implant. RBH at implant placement averaged 7,4 ± 0,4 mm. All patients fulfilled the visiting periods at 12 months. In the control at 24 months, one patient with an implant was lost to follow-up because he moved away. One implant was lost after 12 months of functional loading, due to a failure in the osseointegration. Thirty-two implants fulfilled the survival criteria, representing a 2-year survival rate of 91,6%.

**Table 1:**
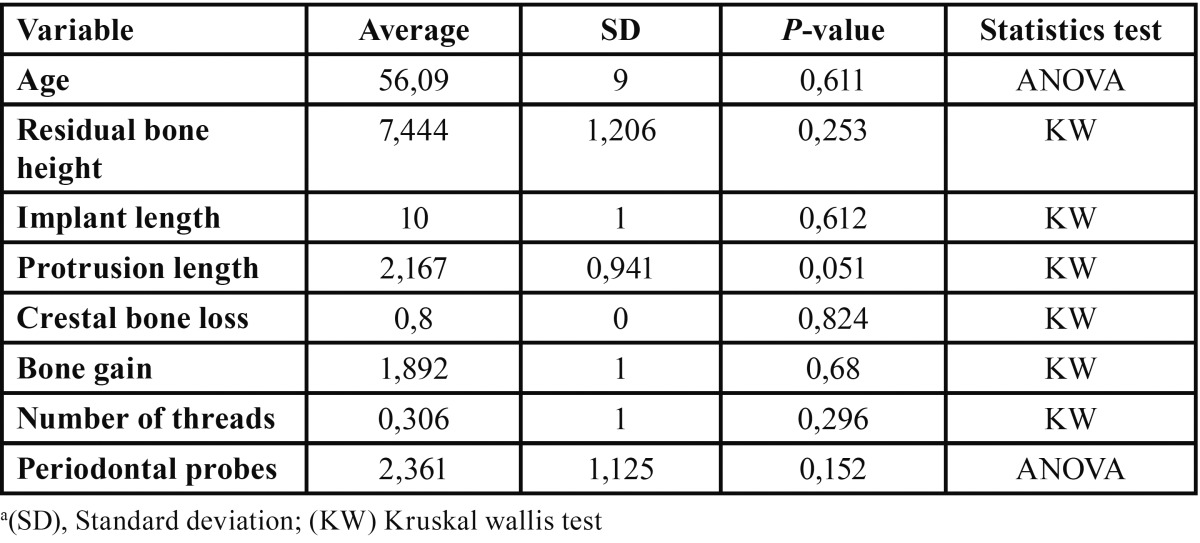
Results of the variables in relation to smoking a. Smoker: 17 yes/ 19 no.

All the patients showed peri-implant bone formation. Mean bone gain was 1,8 ± 0,3 mm. Four implants showed a bone gain exceeding 3 mm. Mean implant protrusion length into the sinus amounted to 2.1 ± 0,3 mm; no implant showed a protrusion length > 5 mm. Mean crestal bone loss amounted to 0,7 ± 0,1 mm.

Regarding the relationship between smoking and periodontal probes, no statistically significant differences were found (Kruskal-Wallis, *P*=0,25), nor does in relation to the number of threads that the implants showed (Kruskal-Wallis, *P*=0,29) or bone gain (One-way ANOVA, *P*=0,79).

None of the four patients that experienced membrane perforation, suffered from any related sinus pathology while during the 2-year follow-up. The four implants sites showed a mean bone gain of 1,9 ± 1,8 mm and a mean crestal bone loss of 0,8 ± 0,4 mm after 24 months.

## Discussion

Due to the inherent characteristics of the posterior maxillary bone, the oral rehabilitation with implants may present some difficulties related to frequent poor quality and insufficient volume of bone found at this area. There have been described two major approaches to overcome these inconvenience by means of elevating sinus membrane: the classical lateral access which consists in the creation of an antrostomy in the anterior wall of the maxillary sinus (16); and the less invasive OSFE technique which uses compression and compaction of the spongy bone of the upper maxilla (17). Both procedures have been performed either with or without the addition of grafting material.

The insertion of grafting material while performing transalveolar sinus floor elevation is a well-known and frequently used procedure to ensure the space between the schneiderian membrane and the floor of sinus cavity for bone regeneration (18). However, the need of bone or biomaterial graftings in this procedure has been questioned. Several studies suggested that the elevation of the schneiderian membrane by itself promotes the bone regeneration by means of the formation of a fibrin clot in the space created. This clot, which is stabilized and protected from external trauma and intra-sinus air pressure, would have the potential to stimulate the bone formation (19,20). This could be confirm by the study of Palma *et al.* (21) in 2006, who in a histological and experimental study in primates showed new bone apposition in contact with schneiderian membrane in coagulum-alone sites, indicating the osteoinductive potential of the membrane. Moreover, the insertion of grafts during the OSFE comes along with some difficulties in the procedure. It has been reported more frequent sinus membrane perforations when grafting material is applied due to the additional pressure that it cause (22). Also the application of grafting material into the sinus cavity by osteotome kit may lead to the risk of increasing the diameter of implant sites (23).

The commonly established guidelines for implant placement in the posterior maxilla mainly consider the residual bone height. The consensus conference (24) held in 1996 on sinus lifting recommended: a classical implant procedure with RBH≥10mm; an osteotome technique in combination with immediate implant placement with RBH=7-9mm; a lateral approach involving a grafting material with immediate or delayed implants placement; and a lateral approach involving bone-grafting material and delayed implant placement with RBH=1-3mm. However recently, with the birth of the new sinus lift techniques the protocols followed in these procedures have been simplified.

Several studies have demonstrated that optimal results in the survival and success of implants rates can be achieved without the use of grafting materials when performing OSFE technique. In that line, Winter *et al.* (25) in 2002 obtained a success rate of 91,4% after 22 months loading in implants placed in an atrophic alveolar ridge with <4mm bone without using bone grafting. Also afterwards, in 2006 Nedir *et al.* (8) in a one-year retrospective study showed a 96% success rate in 25 ITI implants placed in conjunction with no grafting OSFE being the RBH of 5.4±2.3 mm and resulting in only one implant failure. Similar results were found by Lai *et al.* (26) in 2008, yielding a survival rate of 95,2% after a 5 month follow-up, in 42 implants placed with a RBH of 4-8mm (mean 6.4 mm) also using the OSFE technique without graft. The two implants failures of this study were related by the authors to an infection that affected the patient the first week after the surgery. Additionally, Si *et al.* (27) in 2013, showed that performing OSFE in association with grafting materials had no advantages after 3 years´ observation, compared with no grafting OSFE. In the six months immediately after the surgery a meaningful endo-sinus bone gain was found when a graft was applied. However, over time the difference between both groups decreased, reaching the same level of bone gain (3.17 mm±1.95 for the OSFE with graft group and 3.07±1.68 mm for the non-graft OSFE at 36 month follow-up).

The results obtained by these authors are consistent with the present study, in which 36 implants were placed along with an OSFE without graft procedure being the RBH between 4-9mm (averaged 7.4±0.4 mm). Only one implant was failed after 12 months of functional loading due to a peri-implantitis process. After loosing one patient at the last stage of the study, thirty-two implants fulfilled the success criteria defined by Buser et al in 1997, representing a 2-year success rate of 91,6%.

Although more studies seem necessary over the OSFE without grafting, this technique seems efficient and equally predictable as the grafting OSFE. In the present study peri-implant bone formation was obtained in all patients, being the meaning gain of 1.8±0.3 mm ranging from 0.3-4.5mm. This bone gain along with the high survival rates, although in a lesser extent, are in accor-dance with the study of Nedir *et al.* (10) in 2010, who achieved a survival rate of 100% after a 5-year follow-up period over 25 implants and a mean amount of bone gain of 3.2±1.3 mm.

Moreover, implant crestal bone loss recorded in this study (0.7 ± 0.1 mm) is in accordance with the definition of 1mm normal bone loss during the first year, followed by an annual loss <0.2 mm,proposed by Albrektsson *et al.* (28)

Although smoking habit has been widely discouraged when performing oral surgery due to its deleterious effects on the wound healing process, regarding implants or tissue integration (29), and the increased risk of suffering postoperative complications such as infection or peri-implantitis (30), it should not be considered an absolute contraindication for implant treatment. This study has found no significant relationship between tobacco and implant failure, including patients whose daily consumption was more than 10 cigarettes per day. Nevertheless, patients should be advised that they are at greater risk of implant failure if they smoke during the initial healing phase following implant placement and that the interruption of smoking is the best option.

## Conclusions

Implant rehabilitation of edentulous atrophied posterior maxilla can be performed and simplified using the OSFE technique without grafting material. This 2 years study confirms the potential of healing and bone formation of the posterior maxilla below the sinus membrane. Grafting is not seemed to be essential for bone formation in the atrophic maxilla. Implant survival rate was 97,2% while the implant success rate was 91,6%, so the procedure appears to be predictable and sufficient to create bone beyond the natural limit of the sinus allowing treat the compromised posterior maxilla when the RBH is limited with reliable long-term results.

## References

[1] Fugazzotto P (2003). Augmentation of the posterior maxilla: a proposed hierarchy of treatment selection. J Periodontol.

[2] Brägger U, Gerber C, Joss A, Haenni S, Meier A, Hashorva E (2004). Patterns of tissue remodeling after placement of ITI dental implants using an osteotome technique: a longitudinal radiographic case cohort study. Clin Oral Implants Res.

[3] Tatum HJr (1986). Maxillary and sinus implant reconstructions. Dent Clin North Am.

[4] Summers RB (1994). A new concept in maxillary implant surgery: the osteotome technique. Compendium.

[5] Brägger U, Gerber C, Joss A, Haenni S, Meier A, Hashorva E (2004). Patterns of tissue remodeling after placement of ITI dental implants using an osteotome technique: a longitudinal radiographic case cohort study. Clin Oral Implants Res.

[6] Pjetursson BE, Ignjatovi D, Matuliene G, Brägger U, Schmidlin K, Lang NP (2009). Transalveolar maxillary sinus floor elevation using osteotomes with or without grafting material. Part II: radiographic tissue remodeling. Clin Oral Implants Res.

[7] Leblebicioglu B, Ersanli S, Karabuda C, Tosun T, Gokdeniz H (2005). Radiographic evaluation of dental implants placed using an osteotome technique. J Periodontol.

[8] Nedir R, Bischof M, Vazquez L, Szmukler-Moncler S, Bernard JP (2006). Osteotome sinus floor elevation without grafting material: a 1-year prospective pilot study with ITI implants. Clin Oral Implants Res.

[9] Nedir R, Nurdin N, Szmukler-Moncler S, Bischof M (2009). Placement of tapered implants using an osteotome sinus floor elevation technique without bone grafting: 1-year results. Int J Oral Maxillofac Implants.

[10] Nedir R, Nurdin N, Vazquez L, Szmukler-Moncler S, Bischof M, Bernard JP (2010). Osteotome sinus floor elevation technique without grafting: a 5-year prospective study. J Clin Periodontol.

[11] Nedir R, Nurdin N, Khoury P, Perneger T, El Hage M, Bernard JP (2013). Osteotome sinus floor elevation with and without grafting material in the severely atrophic maxilla. A 1-year prospective randomized controlled study. Clin Oral Implants Res.

[12] Fermergård R, Astrand P (2008). Osteotome sinus floor elevation and simultaneous placement of implants--a 1-year retrospective study with Astra Tech implants. Clin Implant Dent Relat Res.

[13] Schmidlin PR, Müller J, Bindl A, Imfeld H (2008). Sinus floor elevation using an osteotome technique without grafting materials or membranes. Int J Periodontics Restorative Dent.

[14] Albrektsson T, Zarb G, Worthington P, Eriksson AR (1986). The long-term efficacy ofcurrently used dental implants: a review and proposed criteria of success. Int J Oral Maxillofac Implant.

[15] Misch CE, Morton LP, Wang HL, Sammartino G, Galindo-Moreno P, Trisi P (2008). Implant success, survival and failure: The International Congress of Oral Implantologists (ICOI) Pisa consensous conference. Implant Dent.

[16] Martos-Díaz P, Naval-Gías L, Sastre-Perez J, González-García R, Bances del Castillo F, Mancha-de la Plata M (2007). Sinus elevation by in situ utilization of bone scrapers: technique and results. Med Oral Patol Oral Cir Bucal.

[17] Calvo-Guirado JL, Saez-Yuguero R, Pardo-Zamora G (2006). Compressive osteotomes for expansion and maxilla sinus floor lifting. Med Oral Patol Oral Cir Bucal.

[18] Tan WC, Lang NP, Zwahlen M, Pjetursson BE (2008). A systematic review of the success of sinus floor elevation and survival of implants inserted in combination with sinus floor elevation. Part II: transalveolar technique. J Clin Periodontol.

[19] Lundgren S, Andersson S, Gualini F, Sennerby L (2004). Bone reformation with sinus membrane elevation: a new surgical technique for maxillary sinus floor augmentation. Clin Implant Dent Relat Res.

[20] Hatano N, Sennerby L, Lundgren S (2007). Maxillary sinus augmentation using sinus membrane elevation and peripheral venous blood for implant-supported rehabilitation of the atrophic posterior maxilla: case series. Clin Implant Dent Relat Res.

[21] Palma VC, Magro-Filho O, De Oliveira JAU, Lundgren S, Salata LA, Sennerby L (2006). Bone reformation and implant integration following maxillary sinus membrane elevation: an experimental study in primates. Clin Implant Dent Relat Res.

[22] Lai HC, Zhuang LF, Lv XF, Zhang ZY, Zhang YX (2010). Osteotome sinus floor elevation with or without grafting: a preliminary clinical trial. Clin Oral Implants Res.

[23] Pjetursson BE, Rast C, Bragger U, Schmidlin K, Zwahlen M, Lang NP (2009). Maxillary sinus floor elevation using the (transalveolar) osteotome technique with or without grafting material. Part I: implant survival and patients’perception. Clin Oral Implants Res.

[24] Jensen OT, Schulman LB, Block MS, Iacone VJ (1998). Report of the sinus consensus conference of 1996. Int J Oral Maxillofac Implants.

[25] Winter AA, Pollack AS, Odrich RB (2002). Placement of implants in the severely atrophic posterior maxilla using localized management of the sinus floor: a preliminary study. Int J Oral Maxillofac Implants.

[26] Lai HC, Zhang ZY, Wang F, Zhuang LF, Liu X (2008). Resonance frequency analysis of stablility on ITI implants with osteotome sinus floor elevation technique without grafting: a 5-month prospective study. Clin Oral implants Res.

[27] Si MS, Zhuang LF, Gu YX, Mo JJ, Qiao SC, Lai HC (2013). Osteotome sinus floor elevation with or without grafting: a 3-year randomized controlled clinical trial. J Clin Periodontol.

[28] Albrektsson T, Zarb GA, Worthington P, Eriksson AR (1986). The long term efficacy of currently used dental implants: A review and proposed criteria of success. Int Jour Oral Maxilofac Implants.

[29] Lindquist LW, Carlsson GE, Jemt T (1997). Association between marginal bone loss around osseointegrated mandibular implants and smoking habits: a 10-year follow-up study. J Dent Res.

[30] Lin TH, Chen L, Cha J, Jeffcoat M, Kao DW, Nevins M (2012). The effect of cigarette smoking and native bone height on dental implants placed immediately in sinuses grafted by hydraulic condensation. Int J Periodontics Restorative Dent.

